# Suppurative Thrombosis of the Portal Vein (Pylephlebits): A Systematic Review of Literature

**DOI:** 10.3390/jcm11174992

**Published:** 2022-08-25

**Authors:** Dorde Jevtic, Tatjana Gavrancic, Ivana Pantic, Terri Nordin, Charles W. Nordstrom, Marina Antic, Nikola Pantic, Marija Kaljevic, Bojan Joksimovic, Milan Jovanovic, Emilia Petcu, Mladen Jecmenica, Tamara Milovanovic, Lawrence Sprecher, Igor Dumic

**Affiliations:** 1 Icahn School of Medicine at Mount Sinai, New York, NY 10029, USA; 2 Department of Internal Medicine, Elmhurst Hospital Center, New York, NY 11373, USA; 3 Department of Hospital Medicine, Mayo Clinic, Jacksonville, FL 32224, USA; 4 Clinic of Gastroenterology and Hepatology, University Clinical Center of Serbia, 11000 Belgrade, Serbia; 5 Mayo Clinic Alix School of Medicine, Rochester, MN 55905, USA; 6 Department of Family Medicine, Mayo Clinic Health System, Eau Claire, WI 54701, USA; 7 Department of Hospital Medicine, Mayo Clinic Health System, Eau Claire, WI 54702, USA; 8 Clinic of Hematology, University Clinical Center of Serbia, 11000 Belgrade, Serbia; 9 School of Medicine, University of Connecticut, Farmington, CT 06269, USA; 10 Faculty of Medicine Foča, University of East Sarajevo, 71123 Sarajevo, Bosnia and Herzegovina; 11 School of Medicine, University of Belgrade, 11000 Belgrade, Serbia; 12 Oceana Gastroenterology Associated, Corona, CA 92881, USA

**Keywords:** pylephlebitis, portal vein thrombosis, suppurative thrombosis of portal vein

## Abstract

Suppurative portal vein thrombosis (pylephlebitis) is an uncommon condition usually associated with an intra-abdominal infection or inflammatory process. In this study, we aimed to synthesize data on previously published cases according to the PRISMA guidelines. A total of 103 patients were included. Patients were more commonly male (71.8%) and had a mean age of 49 years. The most common infection associated with pylephlebitis was diverticulitis (*n* = 29, 28.2%), and *Escherichia coli* was the most isolated pathogen (*n* = 21, 20.4%). Blood cultures were positive in 64 cases (62.1%). The most common site of thrombosis was the main portal vein (PV) in 59 patients (57.3%), followed by the superior mesenteric vein (SMV) in 40 patients (38.8%) and the right branch of the PV in 30 patients (29.1%). Sepsis developed in 60 patients (58.3%). The mortality rate in our review was 8.7%, and independent risk factors for mortality were the presence of pertinent comorbidities (OR 5.5, *p* = 0.02), positive blood cultures (OR 2.2, *p* = 0.02), and sepsis (OR 17.2, *p* = 0.049).

## 1. Introduction

Pylephlebitis, also defined as suppurative thrombophlebitis of the portal vein or its branches, is an uncommon complication of intra-abdominal infection or inflammation (i.e., diverticulitis, appendicitis, pancreatitis), and occurs by the spread of the pathogen through local venules and veins that drain into the portal venous system [1]. The most common microorganisms associated with pylephlebitis are *Bacteroides* spp., *Escherichia coli*, and *Streptococcus* spp. [2]. Pylephlebitis is diagnosed by demonstrating the presence of thrombosis in the portal vein system together with evidence of significant and temporally related infection or inflammation in the abdomen [1,2,3]. Although positive blood cultures are definitive evidence of infection, they are not always required to make a diagnosis, as it is apparent that many patients will not have positive blood cultures despite having infected thrombus in the portal system [1,2,3]. As the disease progresses, patients can develop complications such as sepsis, metastatic abscesses, and multi-organ failure, leading to a mortality rate as high as 19% [2]. Due to the rarity of pylephlebitis, randomized controlled trials and prospective studies describing the risk factors, diagnosis, and, most importantly, the treatment of the disease are lacking and are impractical to conduct. Hence, our systematic review aims to describe patient demographics, risk factors, types and location of thrombosis, types of pathogens, diagnostic modalities, treatment, complications, and outcomes by analyzing data from case reports and case series in a systemic approach as defined by PRISMA (Preferred Reporting Items for Systematic Reviews and Meta-Analyses) guidelines.

## 2. Materials and Methods

We performed a systematic review of the literature following PRISMA guidelines by searching the PubMed/Medline database for articles reporting cases of pylephlebitis using the following search terms: “Pylephlebitis and case report”, “Suppurative portal vein thrombosis and case report”, “Septic portal vein thrombosis and case report”, or “Portal pyemia and case report”. All case reports and case series published from 1 January 2010 to 31 December 2021 were analyzed. We chose to describe the cases dating from 2010, as a prior review previously addressed cases from July 1971 to December 2009 [2].

**Figure 1:**
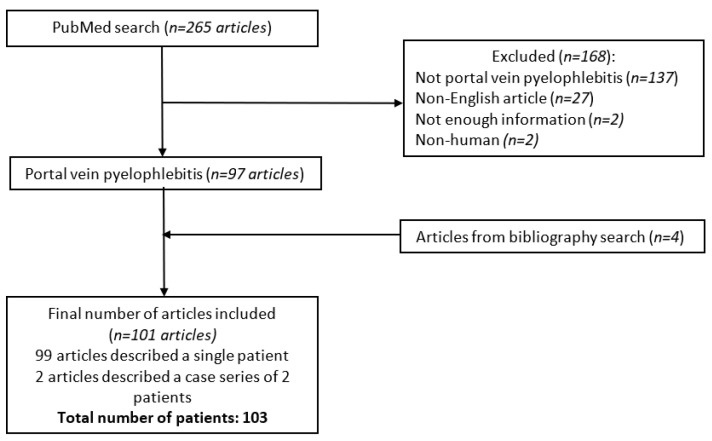
Detailed flowchart of the literature search and article inclusion according to PRISMA guidelines.

Two authors blindly and independently selected the cases (D.J. and I.P.). A senior author (I.D.) made a definitive choice when the two authors did not reach a consensus. All cases with thrombosis of the portal vein or its branches diagnosed by imaging (US, CT, or MRI) and positive blood cultures were included. Additionally, cases with proven portal system thrombosis were included in the absence of positive blood cultures if the clinical presentation was highly suggestive of pylephlebitis. Clinical presentation was considered as “highly suggestive of pylephlebitis” if the cases provided a definite inflammatory and/or infectious intra-abdominal process documented by imaging, together with thrombosis of the portal vein system. Additionally, other extra-abdominal infectious foci had to be ruled out. Articles published in languages other than English; cases where clinical, microbiological, or radiological studies did not support the diagnosis; case reports not providing an adequate amount of information, as well as those reporting non-human subjects were excluded from the analyses. The final number of articles included was 101, which resulted in total number of 103 patients [4,5,6,7,8,9,10,11,12,13,14,15,16,17,18,19,20,21,22,23,24,25,26,27,28,29,30,31,32,33,34,35,36,37,38,39,40,41,42,43,44,45,46,47,48,49,50,51,52,53,54,55,56,57,58,59,60,61,62,63,64,65,66,67,68,69,70,71,72,73,74,75,76,77,78,79,80,81,82,83,84,85,86,87,88,89,90,91,92,93,94,95,96,97,98,99,100,101,102,103,104] (Figure 1).

Following the selection of articles included in the analysis, we created an Excel table to catalog patient demographics, comorbidities, site of infection, signs and symptoms, laboratory data, blood cultures, imaging modalities, thrombosis location, treatment, and outcome.

Pertinent comorbidities were defined as categories of disease that increase the risk of thrombosis and included thrombophilia, hepatocellular carcinoma, cirrhosis, inflammatory bowel disease, intra-abdominal surgery, and pancreatitis. Sepsis was defined by the presence of infection and at least two positive Systemic Inflammatory Response Syndrome criteria [105].

The nonparametric Fisher test and parametric *t*-test for independent samples were used to compare differences between groups for the univariate analysis of risk factors associated with pylephlebitis. A binary logistic regression model was used for the multivariate analysis to assess the association between mortality in patients with pylephlebitis and the multiple variables. All statistical analyses were performed using IBM SPSS Statistics Software version 24.0 for Windows (IBM Corp., Armonk, NY, USA). All *p*-values lower than 0.05 were considered statistically significant.

## 3. Results

### 3.1. Demographic Characteristics

We included 103 patients, of which 74 were male (71.8%), with a mean age of 49 years (range: 20 days to 88 years). There were seven patients under 18 years of age (6.8%). There was no significant difference in pertinent comorbidities, cause of infection, number of isolated bacterial species, or mortality rate between genders (*p* > 0.05). Pertinent comorbidities were present in 29 patients (28.2%), with the most common being intra-abdominal surgery (*n* = 10, 9.7%) (Table 1).

### 3.2. Types of Infection

The most common cause of infection was diverticulitis in 29 patients (28.2%), followed by appendicitis in 20 patients (19.4%) and hepatic abscess in 8 patients (7.8%). Pancreatitis and cholangitis were each present in six patients (5.8%). Blood cultures were positive in 64 patients (62.1%), negative in 21 patients (20.4%), and not reported in 18 patients (17.5%). Monobacterial growth was recovered in 39 patients (37.9%) and polymicrobial growth in 25 patients (24.3%). The most common isolate was *Escherichia coli* in 21 patients (20.4%), followed by *Bacteroides* spp. in 13 patients (12.6%), *Streptococcus* spp. in 12 patients (11.7%), and *Fusobacterium* spp. in 10 patients (9.7%) (see Table 1).

### 3.3. Clinical Presentation

The most common presenting signs and symptoms included fever (*n* = 89, 86.4%) and abdominal pain (*n* = 81, 78.6%). Sepsis was present in 60 patients (58.3%) (see Table 1).

Laboratory abnormalities are reported in Table 1 and most commonly included elevated CRP (*n* = 40/44, 90.9%), leukocytosis (*n* = 78/87, 89.7%), and elevated ESR (*n* = 12/14, 85.7%).

### 3.4. Thrombosis Site and Characteristics

The most common site of thrombosis was the main portal vein (PV) in 59 patients (57.3%), followed by the superior mesenteric vein (SMV) in 40 patients (38.8%), and the right branch of the PV in 30 patients (29.1%). Solitary thrombosis was identified in 54 patients (52.4%), and was found at two sites in 28 patients (27.2%), three sites in 16 patients (15.5%), four sites in 4 patients (2.9%), and five sites in 1 patient (0.9%). In the patient with five sites, thrombosis was diagnosed in the main PV, right PV, left PV, splenic vein, and SMV. Occlusive thrombosis was present in 26 patients (25.2%). Upon follow-up, thrombosis had completely resolved in 25 patients (24.3%), partially resolved in 5 patients (16.5%), persisted in 17 patients (16.5%), and was undocumented in 56 patients (54.3%) (see Table 2). There was no statistically significant association between thrombosis site and outcome of the disease (*p* > 0.05).

### 3.5. Imaging Modalities

The most common imaging modalities used to diagnose patients with pylephlebitis were abdominal US, CT, and MRI, as illustrated in Table 2.

### 3.6. Treatment

Antimicrobial therapy was used in 97 patients (94.2%), with a mean duration of therapy of 25.9 days (range: 1–120 days). Three patients did not receive antimicrobial therapy, and in three cases (2.9%), data were not available. Anticoagulation was used in 79 patients (76.7%), with heparin being the most common (29 patients, 28.2%). The mean duration of anticoagulation was 128.7 days (range 1–365 days). Treatment complications were uncommon, with three patients (2.9%) experiencing bleeding related to anticoagulation, and one patient (0.9%) developing pseudomembranous colitis due to antibiotics.

### 3.7. Complications and Outcome

Complications were reported in 41 patients (38.8%). The most common complication was metastatic abscesses in six patients (5.8%). Bowel ischemia/infarction occurred in four patients (3.9%). Renal failure, pneumonia, and bowel resection each occurred in three patients (2.9%). Cavernous transformation of the portal vein, hemorrhagic shock, portal vein abscess, peritonitis, and multiorgan failure each occurred in two patients (1.9%). Biloma, empyema, respiratory failure, hepatic segment infarction, intraabdominal sepsis, hyperammonemia, and diverticulum perforation each occurred in one patient (0.9%).

The survival rate in our review was 86.4%, with nine patients dying (8.7%) during the disease course. The outcome was not reported in five cases (4.9%). Univariate analysis demonstrated that patients who died had a significantly higher frequency of pertinent comorbidities (*p* = 0.006), a higher mean number of bacteria isolated on blood culture (*p* = 0.009), and a higher rate of sepsis as a complication of disease (*p* = 0.042). Multivariate analysis showed that independent risk factors for mortality were pertinent comorbidities (*p* = 0.021), positive blood cultures (*p* = 0.022), and sepsis (*p* = 0.049). Our review found that patients who develop sepsis have a 17 times greater risk of dying than those who do not (OR = 17.182; *p* = 0.049) (Table 3).

### 3.8. Pediatric Population

In this systematic review, a total of seven pediatric patients with pylephlebitis were identified (three males; median age: 5 months (range: 21 days–17 years)). In two patients, pylephlebitis was associated with umbilical vein catheterization, which makes it the most common cause in this small subgroup of patients. However, it was also reported in cases of liver abscess, following liver transplantation, appendicitis, and enteritis. The most common symptom reported was fever, which occurred in almost all patients (6/7), together with sepsis (4/7). Alterations in complete blood count were most commonly observed and included leukocytosis (6/7) and anemia (5/7). Most patients received antimicrobial (6/7) and anticoagulant treatment (5/7). In two cases, a lethal outcome was reported. 

## 4. Discussion

### 4.1. Epidemiology

Pylephlebitis is an infrequent complication of intra-abdominal infection or inflammatory process (e.g., pancreatitis). Incidence is low and has been reported to range from 0.37 to 2.7 cases per 100,000 person-years [3,106]. A previous systematic review found only 100 case reports of pylephlebitis in non-cirrhotic patients published over four decades between 1971 and 2009 [2]. Interestingly, the number of reported cases has progressively increased over time, with 51 cases reported from 2001 to 2009, and approximately double that number from 2010 to 2022, as described in our review [2]. Although the prior review included only non-cirrhotic cases, we found that cirrhosis is rarely reported, with a rate of 4.9% over the past decade. The higher utilization of imaging methods to detect portal thrombosis combined with greater awareness of the disease has likely led to increased identification of pylephlebitis.

Pylephlebitis affects all genders; however, male patients seem to be affected more frequently. Our review and previously reported studies observed male gender predominance [2,3]. The cause behind this is unclear, particularly as all genders present at a similar age with a similar course, severity, and outcome of the disease. Pediatric patients made up 6.8% of cases in our review, which is significantly lower than the previously described 24% [2].

### 4.2. Microbiology

We observed that positive blood cultures are an independent risk factor for mortality in patients with pylephlebitis. Blood cultures were positive in 62.1% of patients, which is higher than the previously reported rate of 42–44% [2,3]. A higher bacterial load and delay in antibiotic initiation likely contribute to the increased yield of positive cultures. *E. coli* was the most common pathogen isolated on blood culture in 20.4% of patients. Previous studies show conflicting evidence, with *Streptococcus* spp., *Bacteroides* spp., and *E. coli* nearly equally isolated [2,3,106]. *E. coli* is a commensal of the gastrointestinal tract (GIT); therefore, it is not surprising that it commonly causes pylephlebitis given that the source of infection is typically intra-abdominal. Moreover, *E. coli* is the most common pathogen isolated in cases of diverticulitis, and our review has found that this condition most commonly predisposes patients to pylephlebitis [107].

Monobacterial infections were more prevalent, with a frequency of 37.9% in our cohort. It has been postulated that infections by multiple pathogens can have a more severe clinical course and outcome, due in part to the synergistic effect of different species [108]. A recent prospective study failed to demonstrate any difference in clinical course, duration of hospitalization, duration of therapy, and outcome between patients with various polymicrobial and monobacterial intra-abdominal infections [109]. The authors suggested that both types of infections should be treated similarly, with prompt antibiotic initialization having the most significant impact on disease course. Our review did not find that polymicrobial infection was a risk factor for mortality.

It is important to note that blood cultures can often be negative, and this should not rule out the diagnosis of pylephlebitis, especially if signs, symptoms, and imaging suggest it. Furthermore, it is important to note that even if the blood cultures are persistently negative, patients will most likely have other positive cultures, e.g., abscess, ascitic, and peripancreatic fluid [29,58,103]. Nonetheless, blood cultures should be obtained in all patients with a presumptive diagnosis of pylephlebitis.

### 4.3. Clinical Presentation

Typical signs and symptoms that patients present with are the result of intra-abdominal infection and include fever, abdominal pain, diarrhea, vomiting, and nausea. As such, it is challenging to ascertain if symptomatology is due to pylephlebitis or the original infection since both present similarly. In all the cases included in our review, imaging was initially performed to evaluate the infection and help decide on further management. Importantly, in none of the cases did the authors suspect pylephlebitis, and the diagnosis was incidentally discovered.

Our review found that diverticulitis, appendicitis, and hepatic abscess were the most common causes associated with pylephlebitis in decreasing order of frequency. Other studies have also reported diverticulitis as the most common cause, with a frequency of 23.1–30%, similar to our review—28.2% [2,106]. A study by *Choudhry* et al., however, identified pancreatitis as the most common cause in 31% of patients [3]. Regardless of the underlying cause, the mortality rate does not differ.

It is sometimes difficult to differentiate between the causes of pylephlebitis since patients can present with concurrent infectious sites (e.g., colitis and pancreatitis) [14]. One of the most challenging differential diagnoses is a hepatic abscess. In such cases, it is difficult to establish causality. Was the primary process liver abscess with the secondary complication of pylephlebitis, or vice versa? Another infrequently described cause of pylephlebitis is foreign object ingestion. Our review found three cases of a fishbone perforating the intestinal and/or gastric wall, migrating to the liver, and causing pylephlebitis [6,65,103]. Pylephlebitis in these cases usually occurs weeks after the initial ingestion, making it difficult to identify the cause unless a histopathological evaluation of the lesion is performed. Sometimes, a specific presentation of pylephlebitis is described, such as with “the abdominal variant of Lemierre’s syndrome” in cases where the identified pathogen is *Fusobacterium necrophorum* [110]. Finally, the originating infection site may not be evident, and patients might be first diagnosed with pylephlebitis with an infectious source discovered days later, as was the case of a patient in whom appendicitis was diagnosed 14 days after initial presentation [66].

### 4.4. Radiological Findings

CT of the abdomen using intravenous contrast is the best modality to diagnose pylephlebitis [111]. In some cases, thrombosis is not initially evident, and repeat imaging might visualize the thrombus after 48 h [25] or sometimes even two weeks following the development of symptoms [65]. The previous review found that CT was utilized in 51% of patients, while our review found that CT was utilized in 89.3% [2]. In our review, US and MRI were used in 40% and 19% of cases, compared to 35% and 3% in a previous review, respectively [2].

Imaging usually demonstrates intravascular air in the initial stages of pylephlebitis, followed by the presence of thrombi a few days later [111]. Any part of the portal venous system can be affected, and we found that the main PV was the most common site of thrombosis. Other studies demonstrated the right intrahepatic branch as the most common site [3], with the extension of the thrombus from the main PV to the SMV commonly occurring as a complication [2]. Our review did not find that any particular site of thrombosis is associated with increased mortality.

### 4.5. Treatment

Treatment of pylephlebitis consists of broad-spectrum antibiotics to cover both Gram-negative aerobes and anaerobes [2,3], irrespective of the presence of bacteremia. Our study found that antibiotics were used in 94.2% of cases compared to previously reported rates of 91%, 100%, and 100% [1,2,3]. The duration of antibiotic therapy should be at least 6 weeks. In the first 2 weeks, the patient should be treated with parenteral antibiotics, followed by a transition to an oral agent once there is laboratory (normalization or down-trending of leukocytosis and inflammatory markers) and clinical improvement in the patient’s condition. When possible, the final antibiotic regimen should be formulated based on the results of cultures and local antibiogram.

There is no uniform recommendation regarding the use of anticoagulation in the treatment of pylephlebitis. The decision to anticoagulate can be guided by general principles in acute and chronic PV thrombosis without pylephlebitis. Anticoagulation was given more consistently in recent years, being utilized in 76.7% of patients in our study, and 82% in a study by Choudhry et al. [3], compared to previous rates of 35% and 70% [1,2]. In contrast to earlier published results where warfarin was used in 79% of cases [3], our study found that in recent years, various anticoagulation options were prescribed: heparin (28.2%), warfarin (23.3%), low-molecular-weight heparin (23.3%), and Xa inhibitors (11.7%). Previous studies found that anticoagulation can increase the rate of PV thrombosis resolution and recanalization, and can decrease complications of chronic portal hypertension [1,3]. At the same time, there was no difference in the risk of bleeding. Ultimately, our review did not find that providing anticoagulation had a significant mortality benefit (*p* = 0.74), which aligns with prior studies [1].

The mean duration of anticoagulation treatment was 128.7 days, similar to 143 days reported by *Choudhry* et al. [3], indicating that a prolonged course is needed. Two patients received anticoagulation for an indefinite period [14,82]. One was diagnosed with inherited thrombophilia [14], and the other was an HIV-positive patient who continued anticoagulation to prevent noncirrhotic portal hypertension and thrombotic complications [82].

### 4.6. Complications and Outcome

The clinical course of pylephlebitis can be severe, and we found that 58.3% of patients met the criteria for sepsis. This presentation may be evident on admission or may complicate the course of hospitalization. Other studies did not report the incidence of sepsis in their cohort of patients; however, they found sepsis to be among the most common causes of death in patients presenting with pylephlebitis [2,106].

Metastatic abscesses due to the hematogenous dissemination of the pyogenic portal focus were the second most common complication. Our review includes an infant who developed pulmonary abscesses as a complication of pylephlebitis, with their hospital course complicated by respiratory failure, mechanical ventilation, and severe sepsis, culminating in death within a month following presentation [67]. In another case, even though a troublesome focus of brain abscess was diagnosed, the patient had an indolent clinical course and successfully recovered without any complications [7]. Uncommon complications include hemorrhagic shock (due to GIT bleeding), peritonitis, multi-organ failure, biloma, empyema, and intra-abdominal sepsis [24,31,37,57,69,80]. Thrombosis can persist, resolve (entirely or partially), or result in a cavernous transformation of the portal vein (i.e., the formation of veins within or around the previously thrombosed PV) [64,85].

The mortality rate in our review was 8.7%, which is lower than the previously reported mortality rates of 12%, 19%, and 11% [1,2,3]. This decrease is significant and might imply that the identification and treatment of pylephlebitis have improved over time. Sepsis was the cause of death in 88.9% of patients who died, with one patient (11.1%) dying due to pancreatic adenocarcinoma [37]. Our review found that the independent risk factors for mortality in patients suffering from pylephlebitis are positive blood cultures, pertinent comorbidities, and sepsis. These factors increase mortality risk by 2.2-, 5.5-, and 17-fold, respectively. Sepsis is the strongest risk factor for mortality, which reinforces the importance of early aggressive antibiotic management and supportive therapy in this patient population.

Limitations of our study pertain to those of systematic reviews in general, such as publication bias, heterogeneity of data quality from original reports, and retrospective method of research. We included only English-language articles indexed in PubMed/MEDLINE and did not include cases published before 2010. Furthermore, certain laboratory values, presence or absence of occlusive thrombosis, typical follow-up, and resolution of thrombosis are seldom reported in case reports/case series.

## 5. Conclusions

Pylephlebitis is uncommon, rarely reported, and seldom suspected on admission. All genders and any age group can be affected; however, patients with certain comorbidities (cirrhosis, pancreatitis, intra-abdominal surgery, IBD, and thrombophilia) are at increased risk for a more severe disease course and worse outcome. Nearly all patients will have a triad of abdominal pain, fever, and leukocytosis, so practicing clinicians should have a high index of suspicion for pylephlebitis if a patient presents with these signs and symptoms. The diagnostic modality test of choice is abdominal CT, ideally with the use of oral and intravenous contrast. Antibiotics should be used in all patients and should be initiated early, together with intravenous fluids if the patient presents with sepsis. Anticoagulation is usually needed provided the absence of strict contraindications, and appropriate follow-up with imaging is necessary to assess for thrombosis resolution. Sepsis complicates the disease in half of the patients, and it independently increases the risk of mortality 17-fold. As such, prompt management is imperative.

## Figures and Tables

**Table 1:** Demographic data, blood cultures, signs and symptoms, treatments, and outcomes for patients reviewed.

Demographic Characteristics	Number (%)	
*Gender*		
Male	74 (71.8)	
Female	29 (28.2)	
*Pertinent Comorbidities*		
Total	29 (28.2)	
Intra-abdominal surgery	10 (9.7)	
Pancreatitis	8 (7.8)	
Cirrhosis	5 (4.9)	
Thrombophilia	4 (3.9)	
IBD	2 (1.9)	
HCC	1 (0.9)	
**Blood cultures**		
Positive	64 (62.1)	
Negative	21 (20.4)	
Not reported	18 (17.5)	
**Isolated pathogens**		
*E. coli*	21 (20.4)	
*Bacteroides* spp.	13 (12.6)	
*Streptococcus* spp.	12 (11.7)	
*Fusobacterium* spp.	10 (9.7)	
*Staphylococcus* spp.	6 (5.8)	
*Klebsiella* spp.	6 (5.8)	
*Clostridium* spp.	5 (4.8)	
*Enterococcus* spp.	5 (4.8)	
*Candida* spp.	2 (1.9)	
Proteus mirabilis	2 (1.9)	
*Actinomyces* spp.; *Micromonas micros*; *Eggerthella lenta*; *Gemella sanguinis*; *Morganella morganii*	1 (0.9)	
**Sites of infection**		
Diverticulitis	29 (28.2)	
Appendicitis	20 (19.4)	
Hepatic abscess	8 (7.8)	
Pancreatitis	6 (5.8)	
Cholangitis	6 (5.8)	
Cholecystitis; colitis; foreign object ingestion; intra-abdominal surgery	4(3.9)	
Odontogenic bacteria	3 (2.9)	
Perisplenic abscess	2 (1.9)	
Retroperitoneal abscess; jejunitis; bowel ischemia	1 (0.9)	
**Signs and symptoms**		
Fever	89 (86.4)	
Abdominal pain	81 (78.6)	
Sepsis/septic shock	60 (58.3)	
Diarrhea	27 (26.2)	
Vomiting	24 (23.3)	
Jaundice	21 (20.4)	
Anorexia	19 (18.4)	
Nausea	15 (14.6)	
Hepatomegaly	10 (9.7)	
Ascites	8 (7.8)	
Splenomegaly	7 (6.8)	
GI bleeding	5 (4.9)	
**Laboratory findings**		
High CRP	40/44 (90.9)	
Leukocytosis	78/87 (89.7)	
High ESR	12/14 (85.7)	
Hyperbilirubinemia	44/59 (74.6)	
Increased AST, ALT	53/74 (71.6)	
Hypoalbuminemia	11/16 (68.8)	
Thrombocytopenia	19/30 (63.3)	
Anemia	25/42 (59.5)	
**Treatments**		Duration ***
Antimicrobials	97 (94.2)	25.9 (1–120)
Anticoagulation	79 (76.7)	128.7 (1–365)
*Heparin*	29 (28.2)	
*Warfarin*	24 (23.3)	
*LMWH*	24 (23.3)	
*Xa inhibitors*	12 11.7)	
*Antithrombin III*	6 (5.8)	
*Other* ****	5 (4.9)	
*Acenocoumarol*	3 (2.9)	
**Outcome**		
Alive	89 (86.4)	
Dead	9 (8.7)	
Unknown	5 (4.9)	

*** Mean number of days (minimum–maximum). **** Non-specified oral anticoagulants, thrombomodulin alfa, gabexate mesilate, tissue plasminogen activator. IBD, inflammatory bowel disease; HCC, hepatocellular carcinoma; GI, gastrointestinal; CRP, C-reactive protein; ESR, erythrocyte sedimentation rate; AST, aspartate transaminase; ALT, alanine transaminase; LMWH, low-molecular-weight heparin.

**Table 2:** Characteristics of thrombosis in our systematic review.

Imaging	Number (%)
CT	92 (89.3)
US	40 (38.8)
MRI	19 (18.4)
**Site of thrombosis**	
Main PV	59 (57.3)
SMV	40 (38.8)
Right branch PV	30 (29.1)
Left branch PV	25 (24.3)
Splenic vein	13 (12.6)
IMV	10 (9.7)
Umbilical veins	1 (0.9)
**Occlusive thrombosis**	
Yes	26 (25.2)
No	16 (15.6)
Not reported	61 (59.2)
**Thrombosis resolution**	
Yes	25 (24.3)
No	17 (16.5)
Partial	5 (4.9)
Unknown	56 (54.3)

US, ultrasound; CT, computed tomography; MRI, magnetic resonance imaging; PV, portal vein; SMV, superior mesenteric vein; IMV, inferior mesenteric vein.

**Table 3:** The complete list of risk factors and their association with mortality in our systematic review.

Variables	Alive *n* (%) or Mean ± SD	Died *n* (%) or Mean ± SD	Univariate *p*-Value	Multivariate OR	Multivariate *p*-Value
***Gender*** *Female* *Male* ***Age***, *years* ***Pertinent comorbidities*** *No* *Yes* ***Diverticulitis as a cause*** *No* *Yes* ***Appendicitis as a cause*** *No* *Yes* ***Isolated bacteria*** *No* *Yes* ***Number of bacteria isolated*** ***Mono- or polymicrobial infection*** *Monomicrobial* *Polymicrobial* ***Sepsis*** *No* *Yes* ***Number of thrombosis sites*** ***Occlusive thrombosis*** *No* *Yes* ***Anticoagulation*** *No* *Yes*	*26 (29.2)* *63 (70.8)* *49.14 ± 19.57* *68 (76.4)* *21 (23.6)* *63 (70.8)* *26 (29.5)* *71 (79.8)* *18 (20.2)* *17 (23.3)* *56 (76.7)* *1.48 ± 0.68* *35 (62.5)* *21 (37.5)* *13 (20.6)* *50 (79.4)* *1.77 ± 0.95* *14 (36.8)* *24 (63.2)* *8 (10.3)* *70 (89.7)*	*3 (33.3)* *6 (66.7)* *42.39 ± 28.62* *3 (33.3)* *6 (66.7)* *7 (77.8)* *2 (22.2)* *9 (100.0)* *0 (0.0)* *3 (37.5)* *5 (62.5)* *2.40 ± 1.14* *1 (20.1)* *4 (80.0)* *1 (11.1)* *8 (88.9)* *1.44 ± 0.72* *1 (50.0)* *1 (50.0)* *1 (14.3)* *6 (85.7)*	0.796 * 0.348 ** 0.006 * 0.658 * 0.135 * 0.376 * 0.009 ** 0.064 * 0.042 * 0.314 ** 0.708 * 0.740 *	2.428 0.967 5.500 1.598 0.000 2.215 13.784 0.403 17.182 0.512 0.321 1.080	0.258 0.234 **0.021** 0.748 0.998 **0.022** 0.085 0.727 **0.049** 0.533 0.447 0.948

* Fisher test; ** *t*-test for independent samples; OR, odds ratio; significant *p*-values in bold.

## Data Availability

Data are available upon request from corresponding author.
